# It Is Just a Matter of Time: Balancing Homologous Recombination and Non-homologous End Joining at the rDNA Locus During Meiosis

**DOI:** 10.3389/fpls.2021.773052

**Published:** 2021-10-28

**Authors:** Jason Sims, Fernando A. Rabanal, Christiane Elgert, Arndt von Haeseler, Peter Schlögelhofer

**Affiliations:** ^1^Department of Chromosome Biology, Max Perutz Labs, University of Vienna, Vienna BioCenter, Vienna, Austria; ^2^Department of Molecular Biology, Max Planck Institute for Developmental Biology, Tübingen, Germany; ^3^Center for Integrative Bioinformatics Vienna (CIBIV), Max Perutz Labs, University of Vienna and Medical University of Vienna, Vienna BioCenter, Vienna, Austria; ^4^Bioinformatics and Computational Biology, Faculty of Computer Science, University of Vienna, Vienna, Austria

**Keywords:** rDNA, meiosis, homologous recombination, non-homologous end joining, evolution

## Abstract

Ribosomal RNA genes (rDNAs) are located in large domains of hundreds of rDNA units organized in a head-to-tail manner. The proper and stable inheritance of rDNA clusters is of paramount importance for survival. Yet, these highly repetitive elements pose a potential risk to the genome since they can undergo non-allelic exchanges. Here, we review the current knowledge of the organization of the rDNA clusters in *Arabidopsis thaliana* and their stability during meiosis. Recent findings suggest that during meiosis, all rDNA loci are embedded within the nucleolus favoring non-homologous end joining (NHEJ) as a repair mechanism, while DNA repair *via* homologous recombination (HR) appears to be a rare event. We propose a model where (1) frequent meiotic NHEJ events generate abundant single nucleotide polymorphisms and insertions/deletions within the rDNA, resulting in a heterogeneous population of rDNA units and (2) rare HR events dynamically change rDNA unit numbers, only to be observed in large populations over many generations. Based on the latest efforts to delineate the entire rDNA sequence in *A. thaliana*, we discuss evidence supporting this model. The results compiled so far draw a surprising picture of rDNA sequence heterogeneity between individual units. Furthermore, rDNA cluster sizes have been recognized as relatively stable when observing less than 10 generations, yet emerged as major determinant of genome size variation between different *A. thaliana* ecotypes. The sequencing efforts also revealed that transcripts from the diverse rDNA units yield heterogenous ribosome populations with potential functional implications. These findings strongly motivate further research to understand the mechanisms that maintain the metastable state of rDNA loci.

## Introduction

The central importance of the rRNA genes for the biology of any organism is evident, as they are essential for survival and for all cellular processes. They are among the evolutionary oldest and also most highly transcribed genomic regions forming the RNA building blocks of ribosomes. Most eukaryotic genomes contain clusters with hundreds to thousands of rRNA gene copies arranged in tandem which are transcribed and processed within the nucleolus.

In eukaryotes, the 18S, 5.8S, and 25S rRNAs form the scaffold for the small and large ribosomal subunits and all three are encoded together in functional units and transcribed as a single polycistronic 45S precursor transcript by RNA polymerase I (Wallace and Birnstiel, 1966; Moss et al., 2007; Layat et al., 2012). The 45S rRNA gene units (also termed rDNA units) are arranged in a head-to-tail manner in large clusters known as nucleolus organizing regions (NORs; Ritossa and Spiegelman, 1965; Wallace and Birnstiel, 1966). In the *Arabidopsis thaliana* reference ecotype Col-0, the 45S rDNA units are approximately 10kb long and arranged in two clusters, each with ~400 repeats, at the top of chromosomes 2 and 4 (Copenhaver and Pikaard, 1996; Sims et al., 2021). A further component of the large ribosomal subunit, the 5S rRNA, is located on chromosomes 3, 4, and 5 in the *A. thaliana* Col-0 ecotype, also arranged in clusters and transcribed by RNA polymerase III (Murata et al., 1997; Layat et al., 2012). One 45S rDNA unit is also found in proximity of the 5S rDNA located on chromosome 3 (Abou-Ellail et al., 2011). Although rRNA transcripts account for approximately 50% of all transcribed RNAs in a cell, only a fraction of the rRNA genes is transcribed at a given time (Warner, 1999; Grummt and Pikaard, 2003; Pontvianne et al., 2010, 2012).

Recent studies have shown that individual 45S and also 5S rDNA units are not identical. Instead, they display a substantial amount of variability, not only within the intergenic regions but also in the genic regions transcribing the conserved ribosomal RNA subunits (Chandrasekhara et al., 2016; Havlová et al., 2016; Rabanal et al., 2017b). These variants have been exploited as molecular markers to study rDNA cluster-specific expression (see below).

The high level of transcriptional activity leads to torsional stress in rDNA and requires the activity of topoisomerases to relieve the positive and negative torsions (French et al., 2011). During replication, highly transcribed regions of the genome, such as the rDNA loci, may encounter frequent collisions between transcription and replication machineries, which need to be resolved (Castel et al., 2014; García-Muse and Aguilera, 2016; Sims et al., 2021). Both processes mentioned above are sources of DNA damage and genome instability in general. The unique nature of the rDNA loci not only makes them especially vulnerable to various types of DNA damage, it also demands special attention during DNA repair. The highly repetitive rDNA loci, with their hundreds of nearly identical rDNA units arranged head-to-tail, may undergo dramatic re-arrangements during homologous recombination (HR) DNA repair. HR may ultimately lead to lengthening or shortening of the rDNA arrays and in general to copy number instability (Warmerdam et al., 2016). Furthermore, the presence of rDNA clusters on multiple chromosomes adds the additional risk of inter-chromosomal recombination. As outlined in more detail below, during meiosis, a developmental program essential for the recombination of genetic traits, numerous DNA double-strand breaks (DSBs) are introduced. In this context, the rDNA loci are sequestered away from the canonical HR pathway and a different DNA repair pathway is employed, termed non-homologous end joining (NHEJ; Sims et al., 2019). NHEJ will less likely lead to genome re-arrangements and rDNA copy number loss, but may lead to the introduction of single nucleotide polymorphisms (SNPs) and short-range insertions/deletions (InDels; Chang et al., 2017; Wright et al., 2018; Xu and Xu, 2020).

In this review, we summarize the recent findings concerning the stability of the rDNA loci and their inheritance from a perspective of meiosis. We also provide a model, in agreement with the current data, that defines HR and NHEJ as the major determinants of rDNA cluster size and rDNA unit sequence variability.

## Dna Double-Strand Break Formation and Repair

DNA damage, if not appropriately repaired, leads to loss of genetic material, genome re-arrangements, and cell cycle arrest. One of the most deleterious DNA insults are DNA DSBs which can for instance be generated by genotoxic agents or molecular tools like homing endo-nucleases, TALE nucleases, and CRISPR/Cas9 (Wu et al., 2014; Lopez et al., 2021) by endogenous processes like the re-establishment of collapsed replication forks or by a dedicated machinery during meiosis. As mentioned above, DSBs can be repaired by different repair pathways, the most prominent being NHEJ and HR. NHEJ has been found to be active during all cell cycle stages, whereas HR is the dominant repair pathway during S and G2 and is briefly introduced below.

## Non-Homologous End Joining

Non-homologous end joining was first described in mammals, where it is the predominant mechanism for DSB repair in non-cycling, somatic cells. It is differentiated in c-NHEJ (canonical) and a-NHEJ (alternative) pathways, the latter including microhomology-mediated end joining (MMEJ), all with the direct ligation of processed DNA ends as common denominator. Re-joining of blunt ends or ends with a few overlapping bases occurs without regard for preserving the sequence or context integrity (Hays, 2002; McVey and Lee, 2008; Chang et al., 2017).

c-NHEJ is initiated with the recognition and the juxtaposition of the broken ends. In mammals, this step is promoted by the DNA-dependent protein kinase (DNA-PK), a complex composed of the KU heterodimer (Xrcc5/6) and the kinase DNA-PK catalytic subunit (DNA-PKcs; Blackford and Jackson, 2017). It is important to note that DNA-PKcs have not been found to be encoded in plant genomes, indicating that in plants NHEJ is orchestrated differently (Templeton and Moorhead, 2005; Yoshiyama et al., 2013). The Artemis protein and the Xrcc4/DNA ligase IV heterodimer are subsequently recruited, with Artemis involved in the maturation of the DSB ends and the Xrcc4/DNA ligase IV complex catalyzing the resealing of the ends (Lees-Miller and Meek, 2003; Meek et al., 2004; Bleuyard et al., 2006). The KU heterodimer is composed of Ku70 and Ku80 and is involved in recognition, protection, and juxtaposition of the ends of a DSB. DNA-PKcs proteins are recruited to the DSB sites *via* interactions with the Ku/DNA complex and by phosphorylating various substrates (e.g.: Ku70, Ku80, Artemis, Xrcc4; Fell and Schild-Poulter, 2015). Artemis possesses both exo- and endo-nuclease activities and performs phospho-regulated maturation of the DSB ends as it cleaves DNA hairpins and other DNA structures (Lobrich and Jeggo, 2017). The final step, consisting of the ligation of broken ends, is carried out by the Xrcc4/DNA ligase IV heterodimer, which is recruited by DNA-PK. The MRN complex, composed of the proteins Mre11, Rad50, and Nbs1, stimulates this ligase activity *in vitro* and is also implicated in the juxtaposition of the ends of the break (Grawunder et al., 1997; Durdikova and Chovanec, 2017).

The alternative NHEJ pathway MMEJ is promoted in the absence of c-NHEJ factors and involves the alignment of microhomologies at the DSB site (Seol et al., 2018). DNA ends are bound by PARP1 (potentially competing with Ku proteins; Wang et al., 2006; Cheng et al., 2011). Following DNA binding, PARP1 gets activated and poly-ADP-ribosylates itself and various targets in the vicinity leading to more accessible chromatin (Polo and Jackson, 2011; Beck et al., 2014). Subsequently, the MRE11-complex is recruited to process the DNA and prepares them for ligation *via* Ligase I or Ligase III.

The counterparts of most NHEJ proteins have been identified and characterized in plants (Bleuyard et al., 2006; Charbonnel et al., 2011). For instance, LIGASE4 is a well-conserved hallmark factor, also in plants, in the c-NHEJ DNA repair pathway (Friesner and Britt, 2003). MRE11 and its complex partners have also been identified and characterized in plants, and they together are required for both HR and MMEJ. Importantly, no homologs of DNA-PK and Ligase III and some further factors have been identified in plant genomes (Manova and Gruszka, 2015; Yoshiyama, 2016), highlighting some fundamental differences in the DNA damage response in plants and other organisms.

## Homologous Recombination

In contrast, DNA DSB repair *via* the HR pathway preserves sequence integrity. Following DSB formation (see above), initiation of HR depends on the localization of the Mre11-Rad50-Nbs1/Xrs2 (MRN/X) complex and its partner CtIP/Sae2/Com1 to the DSB sites (Wright et al., 2018). The MRN complex bridges the two ends, is involved in DNA end processing, and recruits further processing proteins (e.g., a 5' to 3' exo-nuclease). The nucleolytic activities yield a 3' ssDNA overhang, competent to invade dsDNA to probe for a homologous repair template. In addition, the MRN/X complex recruits the DNA damage kinase ATM/Tel1 which phosphorylates a large number of downstream targets (including Rad9, Rad17, Rad53, Rpa1, Xrs1 Com1/Sae2, and Exo1) involved in DNA repair and checkpoint control (Clerici et al., 2005; Roitinger et al., 2015). The ssDNA ends are coated with the replication protein A (RPA), thereby stimulating the recruitment of recombinases [in yeast *via* Rad52; in higher eukaryotes *via* BRCA2 (Krogh and Symington, 2004)]. The recombinase Rad51 (and in meiosis its relative Dmc1; see below) mediates subsequent strand invasion to probe for homologue sequences, assisted and stimulated by a battery of accessory proteins (Sung et al., 2003; Chan et al., 2019). In S/G2, the cell cycle stage during which HR is promoted, the sister chromatid and the chromatids of the homologous chromosome are available as repair templates. Following invasion and successful homology check, the invading strand is elongated and the displaced strand captured by the ssDNA overhang at the DSB site. Subsequently, the elongated strands are ligated yielding a double holiday junction (dHJ) that can lead, after resolution, to restoration of the original chromosome or to a cross-over and therefore a mutual exchange of chromosome arms. In case the sister chromatid has been used as a repair template, such an exchange is genetically neutral; in case a chromatid of the homologous chromosome has been used, such an exchange yields a chimeric chromosome. The latter is the desired repair product during meiosis to support meiotic chromosome disjunction and increase genetic diversity (Ohkura, 2015).

Alternatively, prior to second-end capture, the recombination intermediate can be dismantled by helicases and the invading, now elongated, strand anneals to the DSB site it originated from (also known as SDSA – synthesis dependent strand annealing). Subsequent DNA synthesis and ligation repairs the lesion, with the potential of some genetic information transfer (gene conversion) in case the template strand contained sequence polymorphisms, but without exchange of chromosome arms.

Different pathways have been identified to dismantle dHJs, utilizing structure-specific resolvases like GEN1, MUS81-EM1, or SLX1-SLX4 (or MLH1/3-EXO1 in meiosis; see also below; San-Segundo and Clemente-Blanco, 2020). Alternatively, dHJs can also be dissolved by a complex containing a helicase (BLM, bloom helicase) and a topoisomerase (TOP3-RMI1), to yield intact, but non-recombined chromosomes (Bizard and Hickson, 2014). HR is a conserved process and plants encode all of the important mediators (Knoll et al., 2014).

In this sense, in canonical non-repetitive regions of the genome, HR delivers a more faithful repair outcome with a high likelihood to re-establish the original DNA sequence, while NHEJ leads mostly to short-range deletions and to some extent to insertions and SNPs (Betermier et al., 2014; Liu and Huang, 2014; Ceccaldi et al., 2016).

## Meiosis

Meiosis is a specific developmental process required for the formation of gametes, carrying the genetic information for the next generation. Meiosis is characterized by two consecutive cell divisions that reduce the genome size by half and by recombination of the paternal and maternal genomes. Novel allelic combinations are created by the mutual exchange of genetic information between parental chromosomes. This depends on meiotic DNA DSBs which are enzymatically induced by the conserved SPO11 protein (together with less conserved partners; Hunter, 2015; Mercier et al., 2015; Robert et al., 2016). About 250–300 DSBs are introduced in each individual meiocyte in Arabidopsis (Edlinger et al., 2011), and they all have to be reliably repaired for successful completion of meiosis. As mentioned, meiotic DSBs are introduced following DNA replication; therefore, cells are in G2-phase with HR being the predominant DNA repair pathway. Meiotic HR is specifically tuned to generate genetic diversity, preferentially using a chromatid of the homologue, and not the sister chromatid, as a repair template [inter-homolog (IH) bias]. Multiple such events along a chromosome ensure that homologous chromosomes recognize each other. At least one IH interaction per chromosome pair has to mature into a cross-over to ensure correct segregation of homologs during the first meiotic division (Gray and Cohen, 2016). In non-repetitive regions, recognition of the homologous partners works very reliably and non-allelic recombination events are not observed. This process is also aided by a meiosis-specific chromosome organization (“bouquet”), clustering telomers (and often also centromeres) to reduce the search space for the ssDNA nucleoprotein filaments (Harper et al., 2004). Genomic loci that are comprised of repetitive sequences, like the rDNA clusters, create a liability during recombination since they can undergo non-allelic exchanges and are a potential source of deletions, duplications, inversions, or translocations (Sasaki et al., 2010).

## Dsb Formation and Repair At the Rdna Locus

Most of the studies concerning DSB repair at the rDNA region involve the use of induced DSBs by exogenous factors or the use of mutants that perturb the stability of the rDNA (Harding et al., 2015; Sluis et al., 2015; Warmerdam et al., 2016). In plants, a recent study employed CRISPR-Cas9 to induce DSBs at the rDNA locus. This led to a large population of plants each containing a varying number of rDNA repeats ranging from about 20 to 200% of the wild-type copy number (Lopez et al., 2021). While these plants represent a powerful resource to study rDNA dynamics in the future, the actual response to the Cas9-mediated DNA lesions has not yet been studied. In mammalian cells, it has been established that the DNA damage response at the rDNA and within the nucleolus depends on a critical threshold: low levels of DSB formation activate NHEJ, excessive DSB formation within the rDNA is repaired *via* HR, concomitant with transcriptional downregulation and nucleolus re-organization (van Sluis and McStay, 2017).

Studying rDNA repair in a meiotic environment is advantageous since a relatively defined number of endogenous DSBs are formed in a tightly regulated fashion. This allows monitoring DSB repair at the rDNA loci under physiological conditions (Sims et al., 2019). In plants, only a handful of factors are known to be involved in the repair process and stability of the rDNA in somatic and meiotic tissues after DSB formation. The RECQ/TOP3/RMI1 complex partner RMI2, the DNA helicases RTEL1, and FANCJ have been shown to be independently needed for maintaining the stability of the 45S rDNA loci in somatic tissues of some plants (Rohrig et al., 2016; Dorn et al., 2019). Furthermore, several additional studies have shown the importance of the chromatin assembly complex CAF-1in preventing DSB formation at the rDNA loci and maintaining rDNA copy numbers (Mozgová et al., 2010; Pavlistova et al., 2016). In addition, low amounts of 45S rDNA copies have shown to promote genomic instability in a genome-wide manner by generating large genomic re-arrangements (Picart-Picolo et al., 2020; Lopez et al., 2021). In meiosis, c/a-NHEJ factors, such as LIG4 and MRE11, have been shown to be important for DNA repair within the rDNA region, whereas HDA6 and NUC2, which are involved in regulating rDNA transcription and nucleolus integrity, are essential for limiting HR at the rDNA (Sims et al., 2019).

## A Balance Between Hr and Nhej

Studies in human cells, employing artificially induced DSBs, have described a re-organization of the nucleolus and a shift from NHEJ repair to HR upon reaching a certain threshold of DNA damage (van Sluis and McStay, 2017). This is concomitant with the formation of the nucleolar caps (Reynolds et al., 1964) and a shutdown of rRNA transcription while breaks in the rDNA persist. Nucleolar caps have not yet been described in other organisms other than humans and mice. In yeast, sites of DSBs within the rDNA re-localize to an extra nucleolar site for repair by HR (Horigome et al., 2019).

Work performed in *A. thaliana* shows that in physiological conditions, such as meiosis, the DNA lesions in the rDNA are preferentially repaired by NHEJ. The nucleolus creates a HR-refractory zone with strongly reduced numbers of HR events at the NORs (Sims et al., 2019). It is anticipated that sporadic events of HR can still occur, and they may leave noticeable traces, like rDNA unit duplication/amplification/loss (copy number variation) and variable numbers of sequence repeats within the 45S rDNA units. Maintaining this unique HR-refractory domain depends on specific chromatin modifications which are distinct from the meiotic nucleus (Sims et al., 2019). In general, an increase in HR at the rDNA locus leads to the loss of units and reduced cell fitness. This is, for instance, well described in the *FAS1* mutant background, in which an HR-dependent shortening of the NORs has been reported (Mozgová et al., 2010; Muchova et al., 2015). Nevertheless, HR events likely also occur in wild-type plants, since a dramatic divergence in rDNA copy numbers is detectable within tens of generations, suggesting the presence of low recurring HR events that lead to a change in NOR length (Rabanal et al., 2017a). Furthermore, the presence of long segments of identical rDNA units is an indication of homogenization events at the rDNA locus, likely mediated by HR repair (Copenhaver and Pikaard, 1996; Sims et al., 2021).

We suggest that DSBs within the rDNA, occurring at physiological levels (e.g., as a results of transcription/replication collisions or generated during the meiotic program), are repaired *via* NHEJ, preserving rDNA unit numbers and unit-internal repeat structures, but at the cost of producing errors. Currently, it is unclear whether there is a preference toward canonical or alternative NHEJ pathways (Sims et al., 2019). However, there are indications that both pathways are necessary for repairing lesions within the rDNA since LIG4 and MRE11 have an equal impact on rDNA stability. It is important to mention that DSBs generated within the rDNA by transcription/replication conflicts or during meiosis are still rare events (Sims et al., 2019).

In general, the errors produced by the NHEJ pathways have the potential to generate sequence diversity between the rDNA units, and one would expect for them to accumulate at the transcriptional start and termination sites of each unit, due to selection against mutations in the portions of the rDNA that yield rRNA integrated into ribosomes. In fact, the highest number of SNPs and InDels is found in the external transcribed sequences (ETS), particularly close to the promoter and terminator regions (Chandrasekhara et al., 2016; Rabanal et al., 2017b). In contrast, very few SNPs/InDels are found within the portions transcribing the ribosomal RNA subunits (18S, 5.8S and 25S). This correlates well with the suggested high levels of transcriptional stress in the rDNA, with purifying selection acting on the regions transcribing rRNA subunits and with DNA lesions being repaired *via* NHEJ.

**Figure 1:**
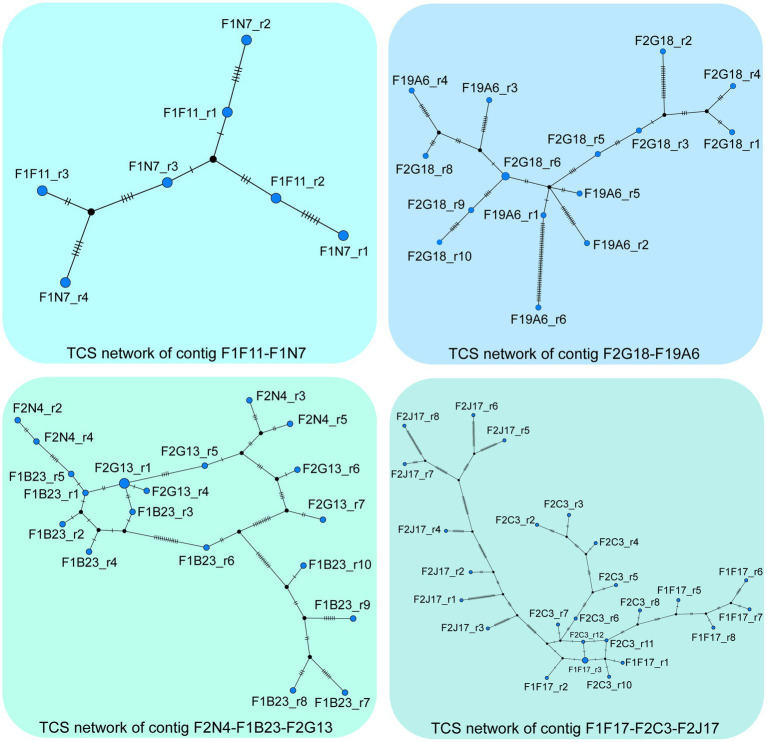
The TCS networks were inferred from rDNA units of the contigs F2N4-F1B23-F2G13, F1F17-F2C3-F2J17, F1F11-F1N27 and F2G18-F19A6 identified in (Sims et al., 2021). The first unit of BAC F2N4 and the fourth unit of BAC F1F11 were excluded from the analysis. Furthermore, the highly repetitive *Sal*I boxes were not taken into account for this data analysis. In the network, each node represents a unique sequence and its size is proportional to its frequency within the data. Short vertical bars on the lines connecting similar sequences represent the number of variations between them. Visualizations of the analyses of the 59 individual BACs are available upon request.

Imbalanced accumulation of polymorphisms is also apparent between the two NORs of *A. thaliana*. A recent study combining long- and short-read sequencing technologies to define the nucleotide composition and organization of 405 individual rDNA units of NOR2 of ecotype Col-0 identified less SNPs/InDels on the transcriptionally less active NOR2, than on NOR4 (Sims et al., 2021). To display the sequence diversity of these 405 rDNA units, we utilized their data and generated a phylogenetic network. For the analysis, we excluded the highly repetitive region of the *Sal*I repeat boxes from each unit. The TCS network was inferred (Supplementary Figure S1) using the integrated method of the TCS approach (Templeton et al., 1992; Clement et al., 2002), which is based on the concept of statistical parsimony in PopArt (Leigh and Bryant, 2015). The network shows a lack of phylogenetic structure in the data indicating that a lot of parallel and reverse mutations obscure the relations between the units and that the conservative nature of the rDNA units in general may possibly mask local phylogenetic information. To address this latter point, we repeated the TCS analysis for short stretches of rDNA units (represented in the 59 BACs as published in Sims et al., 2021). Indeed, the majority of the BACs show a clear tree-like structure, with only very little reticulation. Thus, locally, the evolutionary process follows a classical tree-like pattern. Moreover, directly adjacent units on a BAC tend to be next to each other in the tree (data and visualization available upon request). The contigs identified in (Sims et al., 2021) provide additional evidence of tree-like evolution of the NOR2 region (Figure 1). Though the tree-like relation breaks down, the more rDNA units are analyzed due to multiple identical units occurring along the NOR2 region.

A plausible explanation for the higher abundance of SNPs/InDels on NOR4 could be derived from the fact that in the ecotype Col-0, NOR4 is transcriptionally active in all analyzed tissues, while NOR2 is selectively silenced during development, and it is only active in certain tissue types (Chandrasekhara et al., 2016; Rabanal et al., 2017a). Transcriptional stress *per se* is a prime source of DSBs, and the rDNA is considered a hotspot of transcription and replication stress (Takeuchi et al., 2003). Since the pattern of NOR expression varies greatly among Arabidopsis ecotypes with some expressing predominantly one and some the other NOR (and some both), it would be interesting to analyze whether rDNA polymorphisms are positively correlated with transcriptional activity in different ecotypes.

It is interesting to speculate that the nucleolus represents an HR-refractory sub-compartment within the nucleus during meiosis (and after pre-meiotic DNA replication). As stated above, both NORs are transcriptionally activated in order to be recruited to the nucleolus and embedded in its HR-refractory zone (Sims et al., 2019). Perturbing the rDNA transcriptional activity or the nucleolar architecture generates an imbalance in the rDNA protective mechanism. In this sense, rDNA transcriptional activation, and subsequent recruitment into the nucleolus, could be a key regulatory mechanism to determine the mode of rDNA repair after DNA damage. The recruitment into the nucleolus following transcription is a conserved feature of rDNA (Pontvianne et al., 2013; Sims et al., 2019).

The protective mechanisms surrounding the 45 rDNA regions could not be limited to the nucleolus itself, since in certain tissues, the majority of 45S rDNA genes are not transcribed and excluded from the nucleolus. Inactive NOR4 rDNA genes are generally located at the nucleolar periphery, whereas NOR2 rDNA genes are completely excluded from the nucleolus area (Pontvianne et al., 2013).

It remains unknown whether the nucleolus plays a protective role in other plant tissues or in other organisms. In human cells, massive DNA damage of the rDNA leads to the formation of nucleolar caps. It has been shown that these caps contain broken rDNA which then becomes available to the HR machinery of the nucleus (Sluis et al., 2015), lending support to the idea that the nucleolus represents a general and conserved HR-refractory sub-compartment. Hence, the nucleolus might have the intrinsic property of excluding HR-related proteins. In line with this idea, the nucleolar proteomes of *Arabidopsis* and of humans showed no evidence of the presence of HR proteins (Andersen et al., 2005; Montacié et al., 2017).

## Controlling Sequence Homogeneity and Heterogeneity

The repetitive rDNA loci are considered intrinsically unstable genomic regions since they are prone to various types of DNA damage and repair events. The sequence variations identified in individual rDNA units (Chandrasekhara et al., 2016; Havlová et al., 2016; Rabanal et al., 2017b; Sims et al., 2021) may represent past DNA repair events following an error-prone pathway (NHEJ). Taking into consideration rDNA copy numbers, it is possible to evaluate the history of DNA repair events following an error-free pathway (HR). While sequence variations of rDNA units can readily be analyzed in individual plants, the evaluation of rDNA unit copy number variations demands the analysis of large populations or multiple successive generations (Rabanal et al., 2017b; Sims et al., 2021).

The rDNA copy number can also be considered as a genetic trait and studied in pedigrees. Indeed, when analyzing the trait of “rDNA copy number” over a few generations (two generations in F2s, about eight in recombinant inbred lines – RILs), it appears stable enough that it can be mapped to either NOR in segregating populations. Moreover, the apparent lack of F1-like rDNA copy number phenotypes after several generations of inbreeding in a RIL population further strengthens the notion that the NORs of homologous chromosomes rarely recombine, in agreement with the idea that the nucleolus is a HR-refractory sub-compartment of the nucleus. Importantly, analyzing a wider generational time window, a progressive divergence in the number of rDNA units in single seed descent *A. thaliana* plants was apparent within tens of generations. As a consequence of this unstable inheritance, and in spite of the fact that rDNA unit numbers vary considerably in natural *A. thaliana* populations (Davison et al., 2007; Long et al., 2013), genome-wide association studies failed to map the source of the variation to either of the NORs (Long et al., 2013). This means that rare events of HR might take place, only evident in large populations or when observing multiple successive generations, which lead to dramatic rDNA unit number variations.

In contrast, within plants containing a small amount of 45S rDNA units, the rDNA gene copy numbers can be quickly restored and amplified to wild-type levels. This indicated that there is mechanism in place to restore the 45S rDNA copy numbers within individuals with low amount of rRNA genes (Pavlistova et al., 2016).

## Functional and Evolutionary Impact of Rdna Heterogeneity

Different studies on different organisms (including humans, flies, worms, and plants) have shown that the rDNA genes are not identical either within or among individuals of the same species (Gonzalez et al., 1985; Keller et al., 2006; Stage and Eickbush, 2007; Pillet et al., 2012; Bik et al., 2013). Nevertheless, certain SNPs/Indels are stable and abundant enough in either of the two NORs in *A. thaliana* that they qualify to serve as reporters of NOR-specific expression (Chandrasekhara et al., 2016; Rabanal et al., 2017a). There is unequivocal evidence of selective silencing of one of the two NORs during vegetative development in *A. thaliana*, with the majority of all rRNAs being generated just from one locus. Nevertheless, there is also compelling evidence that (1) there is selective transcriptional activation of certain rDNA units from the otherwise silenced NOR locus in some tissues and (2) that not all rDNA units at the active NOR locus are transcribed at the same time (Pontvianne et al., 2013). rDNA unit variants are not randomly distributed along the NORs [at least established for NOR2 (Sims et al., 2021)], but rather in variant sub-clusters that share certain SNPs/Indels combinations. In some instances, these blocks of corresponding rDNA units are disrupted by rDNA units of a different subtype, but still are transcriptionally co-regulated (Sims et al., 2021). These findings provide a solid base for the future dissection of the fine-tuned regulation of expression of rDNA variant units within a NOR.

It is tempting to speculate that the heterogeneous population of rDNA units and their regulated expression has an important impact on protein translation. The presence of expressed rRNA variants has been shown in various organisms by analyzing total RNA (Kuo et al., 1996; Carranza et al., 1999; Tseng et al., 2008; Rabanal, Mandáková, et al., 2017; Simon et al., 2018). Some of the identified SNPs/InDels were located within the genic regions that encode the 25S and 18S rRNA subunits which are integrated into ribosomes. Furthermore, several studies demonstrated that variant rRNAs are incorporated into polysomes, the ribosomal fraction actively committed to protein translation (Gonzalez et al., 1988; Cloix et al., 2002; Mentewab et al., 2011; Dimarco et al., 2012; Kurylo et al., 2018; Parks et al., 2018; Sims et al., 2021). Interestingly, various of these rRNA gene variants are differentially expressed in a tissue-specific manner. Furthermore, some sequence variations are located in regions that could have a functional impact on the biology of ribosomes. Most of the genic rRNA sequence variations are located in ribosomal expansion segments, that vary greatly between species, but could have an important impact on interacting proteins. A few SNPs/InDels occur in the rRNA core domains. For instance, one G to T transition present in *A. thaliana* is located between the H74 and H88 ribosomal domains at the peptidyl transferase site and thus has the potential to impact ribosomal translation directly. In the parasite *Plasmodium*, two structurally distinct 18S rRNAs are differentially expressed during its life cycle (Gunderson et al., 1987; Waters et al., 1989). And more recently, the expansion segment 9S has been shown to selectively recruit *Hox9* mRNA *via* its 5' UTR stem-loop (Leppek et al., 2020).

In addition, it has been shown that in the bacteria *Vibrio vulnificus*, from a heterogeneous population of ribosomes, it primarily uses ribosomes containing a particular ribosomal RNA variant to translate stress-related mRNA (Song et al., 2019; Leppek et al., 2020). Similarly, in *Escherichia coli*, a specific branch of the stress response utilizes a truncated rRNA to selectively bias translation of stress response proteins (Vesper et al., 2011).

## Conclusions/Perspectives

The most current sequencing technologies, in combination with detailed and large-scale population studies and in-depth analyses of ribosomal RNA variants, have generated novel and exciting insights.

**Figure 2:**
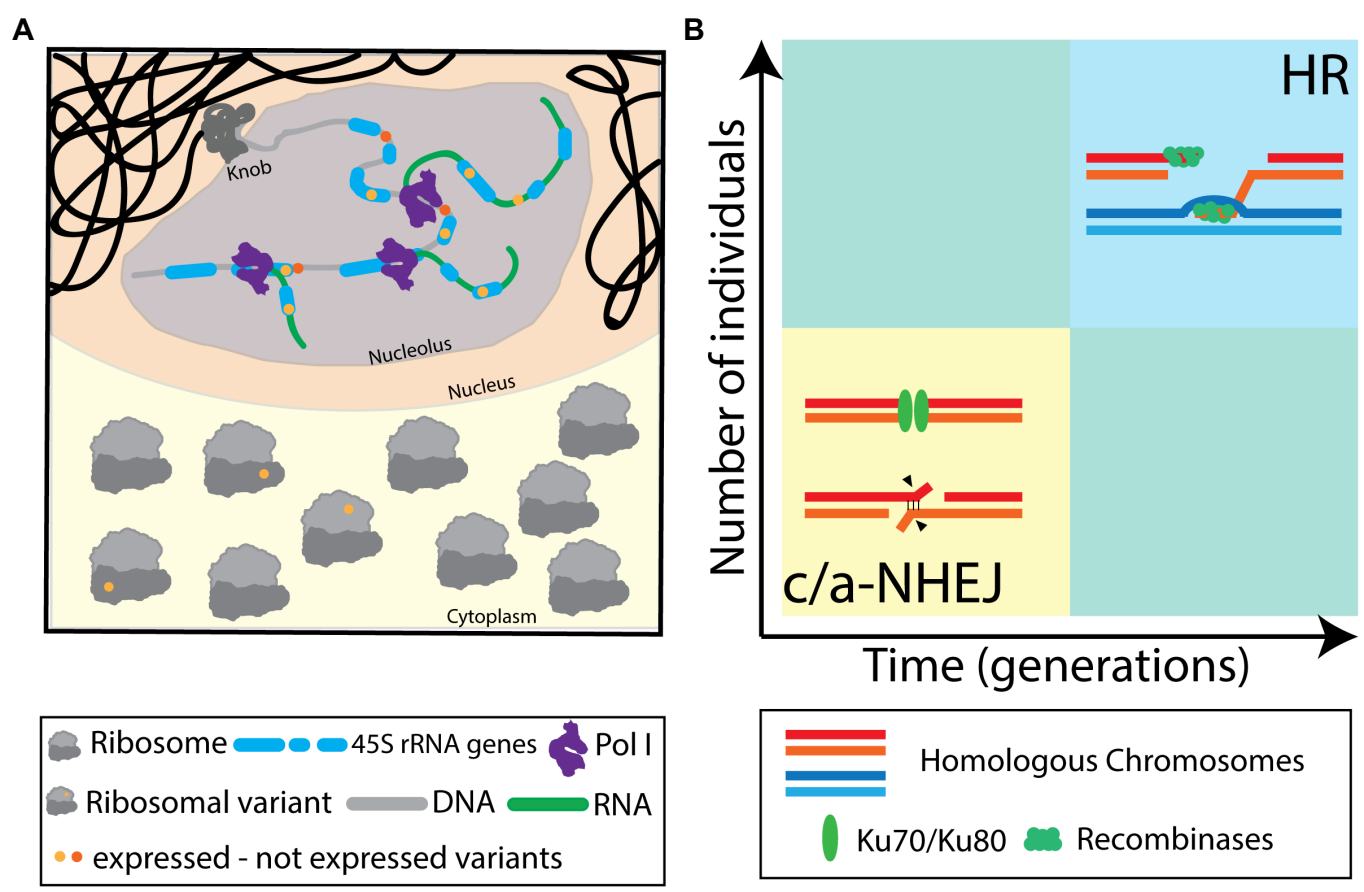
**(A)** Illustration of transcription of variant rRNAs from non-identical 45 rDNA units and their integration into translating ribosomes. The concept of heterogeneous ribosomes has been introduced considering different protein compositions. Here, this concept is extended, also considering different rRNA variants. **(B)** Diagram illustrating the occurrence of non-homologous end joining (NHEJ) and homologous recombination (HR) as DNA repair modes in the highly repetitive nucleolus organizing regions (NORs) during meiosis. NHEJ is considered to be the commonly deployed repair pathway, leading to short-range repair scars in the affected rDNA units, contributing to sequence heterogeneity and preserving the integrity of the NOR. Meiotic DNA repair events *via* HR are considered rare events and will only be evident in large populations, over multiple generations. HR may contribute to NOR size variability and rDNA unit homogenization.

Without any doubt, the NORs cannot be regarded as stable, rigid domains comprised of – nearly – identical rDNA units anymore, but rather as dynamic chromosomal loci with high variation in rDNA unit copy numbers and sequences. We consider a delicate balance of the HR and NHEJ DNA repair mechanisms to be responsible for the dynamic nature of the NORs. We suggest that frequent (meiotic) NHEJ events generate abundant SNPs and InDels within the rDNA, resulting in a heterogeneous population of rDNA units. We also propose that rare HR events dynamically change rDNA unit numbers. The latter may only be observed in large populations and/or over many generations (Figure 2).

Furthermore, the ribosomes are no longer seen as invariant machines that translate proteins from available mRNAs but rather as a heterogeneous population of ribonuclear complexes, differing in rRNA and protein composition, with defined functions controlling protein translation (Figure 2).

In the future, it will be interesting to generate the detailed sequence information of NORs from various organisms, ecotypes, and individuals. Knowledge of the precise rDNA unit sequences will allow detailed analyses of the dynamic changes of the NORs, their (potentially context dependent) differential transcriptional regulation, and the integration of rRNA variants into actively translating ribosomes (with the potential to impact protein translation).

## Author Contributions

JS, FR, and PS: conceptualization. JS and CE: data analysis and visualization. PS, AH, and FR: funding acquisition. All authors contributed to the writing of the article and approved the submitted version.

## Funding

We thank the Austrian Science Fund FWF (SFB F34, DK W1238-B20, I 3685-B25 to PS; W1207-B09 to AH), the European Union (FP7-ITN 606956 to PS), the Human Frontiers Science Program (HFSP Long-Term Fellowship LT000819/2018-L to FR), and the Max Planck Society (FR) for funding.

## Conflict of Interest

The authors declare that the research was conducted in the absence of any commercial or financial relationships that could be construed as a potential conflict of interest.

## Publisher’s Note

All claims expressed in this article are solely those of the authors and do not necessarily represent those of their affiliated organizations, or those of the publisher, the editors and the reviewers. Any product that may be evaluated in this article, or claim that may be made by its manufacturer, is not guaranteed or endorsed by the publisher.

## Supplementary Material

**Supplementary Figure S1 | ** TCS network was inferred from all sequences of the rDNA units of 59 BACs without the region of the highly repetitive *Sal*I boxes. To build the network, the integrated method of the TCS approach, which is based on the concept of statistical parsimony, in PopArt was used. In the network, each node represents a unique sequence, its size is proportional to its frequency within the data, and its color indicates from which BAC a rDNA originated from. In other words, a big, multicolored node is a collection of different rDNA units which are identical and come from different BACs (and therefore different locations within the NOR2). Short vertical bars on the lines connecting similar sequences represent the number of variations between them. Furthermore, sequences, not present in the data, were inferred and represented as small black dots. The TCS network contains 39 nodes which comprise more than one rDNA unit. In total, 238 rDNA units occur in the 39 nodes. The biggest node includes 62 rDNA units.

## References

[ref1] Abou-Ellail M. Cooke R. Sáez-Vásquez J. (2011). Variations in a team: major and minor variants of *Arabidopsis thaliana* rDNA genes. Nucleus 2, 294–299. doi: 10.4161/nucl.2.4.16561, PMID: 21941113

[ref2] Andersen J. S. Lam Y. W. Leung A. K. L. Ong S.-E. Lyon C. E. Lamond A. I.. (2005). Nucleolar proteome dynamics. Nature 433, 77–83. doi: 10.1038/nature03207, PMID: 15635413

[ref3] Beck C. Robert I. Reina-San-Martin B. Schreiber V. Dantzer F. (2014). Poly(ADP-ribose) polymerases in double-strand break repair: focus on PARP1, PARP2 and PARP3. Exp. Cell Res. 329, 18–25. doi: 10.1016/j.yexcr.2014.07.003, PMID: 25017100

[ref4] Betermier M. Bertrand P. Lopez B. S. (2014). Is non-homologous end-joining really an inherently error-prone process? PLoS Genet. 10:e1004086. doi: 10.1371/journal.pgen.1004086, PMID: 24453986 PMC3894167

[ref5] Bik H. M. Fournier D. Sung W. Bergeron R. D. Thomas W. K. (2013). Intra-genomic variation in the ribosomal repeats of nematodes. PLoS One 8:e78230. doi: 10.1371/journal.pone.0078230, PMID: 24147124 PMC3795665

[ref6] Bizard A. H. Hickson I. D. (2014). The dissolution of double Holliday junctions. Cold Spring Harb. Perspect. Biol. 6:a016477. doi: 10.1101/cshperspect.a016477, PMID: 24984776 PMC4067992

[ref7] Blackford A. N. Jackson S. P. (2017). ATM, ATR, and DNA-PK: the trinity at the heart of the DNA damage response. Mol. Cell 66, 801–817. doi: 10.1016/j.molcel.2017.05.015, PMID: 28622525

[ref8] Bleuyard J.-Y. Y. Gallego M. E. White C. I. (2006). Recent advances in understanding of the DNA double-strand break repair machinery of plants. DNA Repair 5, 1–12. doi: 10.1016/j.dnarep.2005.08.017, PMID: 16202663

[ref9] Carranza S. Baguñà J. Riutort M. (1999). Origin and evolution of paralogous rRNA gene clusters within the flatworm family Dugesiidae (Platyhelminthes, Tricladida). J. Mol. Evol. 49, 250–259. doi: 10.1007/PL00006547, PMID: 10441676

[ref10] Castel S. E. Ren J. Bhattacharjee S. Chang A. Y. Sánchez M. Valbuena A.. (2014). Dicer promotes transcription termination at sites of replication stress to maintain genome stability. Cell 159, 572–583. doi: 10.1016/j.cell.2014.09.031, PMID: 25417108 PMC4243041

[ref11] Ceccaldi R. Rondinelli B. D’Andrea A. D. (2016). Repair pathway choices and consequences at the double-strand break. Trends Cell Biol. 26, 52–64. doi: 10.1016/j.tcb.2015.07.009, PMID: 26437586 PMC4862604

[ref12] Chan Y. L. Zhang A. Weissman B. P. Bishop D. K. (2019). RPA resolves conflicting activities of accessory proteins during reconstitution of Dmc1-mediated meiotic recombination. Nucleic Acids Res. 47, 747–761. doi: 10.1093/nar/gky1160, PMID: 30462332 PMC6344864

[ref13] Chandrasekhara C. Mohannath G. Blevins T. Pontvianne F. Pikaard C. S. (2016). Chromosome-specific NOR inactivation explains selective rRNA gene silencing and dosage control in Arabidopsis. Genes Dev. 30, 177–190. doi: 10.1101/gad.273755.115, PMID: 26744421 PMC4719308

[ref14] Chang H. H. Y. Pannunzio N. R. Adachi N. Lieber M. R. (2017). Non-homologous DNA end joining and alternative pathways to double-strand break repair. Nat. Rev. Mol. Cell Biol. 18, 495–506. doi: 10.1038/nrm.2017.48, PMID: 28512351 PMC7062608

[ref15] Charbonnel C. Allain E. Gallego M. E. White C. I. (2011). Kinetic analysis of DNA double-strand break repair pathways in Arabidopsis. DNA Repair 10, 611–619. doi: 10.1016/j.dnarep.2011.04.002, PMID: 21530420

[ref16] Cheng Q. Barboule N. Frit P. Gomez D. Bombarde O. Couderc B.. (2011). Ku counteracts mobilization of PARP1 and MRN in chromatin damaged with DNA double-strand breaks. Nucleic Acids Res. 39, 9605–9619. doi: 10.1093/nar/gkr656, PMID: 21880593 PMC3239177

[ref17] Clement M. Snell Q. Walke P. Posada D. Crandall K. (2002). “TCS: estimating gene genealogies.” in *Proceedings of the 16th International Parallel Distributed Processing Symposium*; April 15-19, 2002; Lauderdale, FL, USA, 184.

[ref18] Clerici M. Mantiero D. Lucchini G. Longhese M. P. (2005). The *Saccharomyces cerevisiae* Sae2 protein promotes resection and bridging of double strand break ends. J. Biol. Chem. 280, 38631–38638. doi: 10.1074/jbc.M508339200, PMID: 16162495

[ref19] Cloix C. Tutois S. Yukawa Y. Mathieu O. Cuvillier C. Espagnol M.-C.. (2002). Analysis of the 5S RNA pool in *Arabidopsis thaliana*: RNAs are heterogeneous and only two of the genomic 5S loci produce mature 5S RNA. Genome Res. 12, 132–144. doi: 10.1101/gr.181301, PMID: 11779838 PMC155267

[ref20] Copenhaver G. P. Pikaard C. S. (1996). Two-dimensional RFLP analyses reveal megabase-sized clusters of rRNA gene variants in *Arabidopsis thaliana*, suggesting local spreading of variants as the mode for gene homogenization during concerted evolution. Plant J. 9, 273–282. doi: 10.1046/j.1365-313X.1996.09020273.x, PMID: 8820611

[ref21] Davison J. Tyagi A. Comai L. (2007). Large-scale polymorphism of heterochromatic repeats in the DNA of *Arabidopsis thaliana*. BMC Plant Biol. 7:44. doi: 10.1186/1471-2229-7-44, PMID: 17705842 PMC2000876

[ref22] Dimarco E. Cascone E. Bellavia D. Caradonna F. (2012). Functional variants of 5S rRNA in the ribosomes of common sea urchin *Paracentrotus lividus*. Gene 508, 21–25. doi: 10.1016/j.gene.2012.07.067, PMID: 22967708

[ref23] Dorn A. Feller L. Castri D. Röhrig S. Enderle J. Herrmann N. J.. (2019). An Arabidopsis FANCJ helicase homologue is required for DNA crosslink repair and rDNA repeat stability. PLoS Genet. 15:e1008174. doi: 10.1371/journal.pgen.1008174, PMID: 31120885 PMC6550410

[ref24] Durdikova K. Chovanec M. (2017). Regulation of non-homologous end joining via post-translational modifications of components of the ligation step. Curr. Genet. 63, 591–605. doi: 10.1007/s00294-016-0670-7, PMID: 27915381

[ref25] Edlinger B. Schlogelhofer P. Schlögelhofer P. Schlogelhofer P. Schlögelhofer P. (2011). Have a break: determinants of meiotic DNA double strand break (DSB) formation and processing in plants. J. Exp. Bot. 62, 1545–1563. doi: 10.1093/jxb/erq421, PMID: 21220780

[ref26] Fell V. L. Schild-Poulter C. (2015). The Ku heterodimer: function in DNA repair and beyond. Mutat. Res. Rev. Mutat. Res. 763, 15–29. doi: 10.1016/j.mrrev.2014.06.002, PMID: 25795113

[ref27] French S. L. Sikes M. L. Hontz R. D. Osheim Y. N. Lambert T. E. El Hage A.. (2011). Distinguishing the roles of topoisomerases I and II in relief of transcription-induced torsional stress in yeast rRNA genes. Mol. Cell. Biol. 31, 482–494. doi: 10.1128/MCB.00589-10, PMID: 21098118 PMC3028620

[ref28] Friesner J. Britt A. B. (2003). Ku80- and DNA ligase IV-deficient plants are sensitive to ionizing radiation and defective in T-DNA integration. Plant J. 34, 427–440. doi: 10.1046/j.1365-313X.2003.01738.x, PMID: 12753583

[ref29] García-Muse T. Aguilera A. (2016). Transcription-replication conflicts: how they occur and how they are resolved. Nat. Rev. Mol. Cell Biol. 17, 553–563. doi: 10.1038/nrm.2016.88, PMID: 27435505

[ref30] Gonzalez I. L. Gorski J. L. Campen T. J. Dorney D. J. Erickson J. M. Sylvester J. E.. (1985). Variation among human 28S ribosomal RNA genes. Proc. Natl. Acad. Sci. U. S. A. 82, 7666–7670. doi: 10.1073/pnas.82.22.7666, PMID: 3865188 PMC391394

[ref31] Gonzalez I. L. Sylvester J. E. Schmickel R. D. (1988). Human 28S ribosomal RNA sequence heterogeneity. Nucleic Acids Res. 16, 10213–10224. doi: 10.1093/nar/16.21.10213, PMID: 3194198 PMC338847

[ref32] Grawunder U. Wilm M. Wu X. Kulesza P. Wilson T. E. Mann M.. (1997). Activity of DNA ligase IV stimulated by complex formation with XRCC4 protein in mammalian cells. Nature 388, 492–495. doi: 10.1038/41358, PMID: 9242410

[ref33] Gray S. Cohen P. E. (2016). Control of meiotic crossovers: from double-strand break formation to designation. Annu. Rev. Genet. 50, 175–210. doi: 10.1146/annurev-genet-120215-035111, PMID: 27648641 PMC5319444

[ref34] Grummt I. Pikaard C. S. (2003). Epigenetic silencing of RNA polymerase I transcription. Nat. Rev. Mol. Cell Biol. 4, 641–649. doi: 10.1038/nrm1171, PMID: 12923526

[ref35] Gunderson J. H. Sogin M. L. Wollett G. Hollingdale M. de la Cruz V. F. Waters A. P.. (1987). Structurally distinct, stage-specific ribosomes occur in Plasmodium. Science 238, 933–937. doi: 10.1126/science.3672135, PMID: 3672135

[ref36] Harding S. M. Boiarsky J. A. Greenberg R. A. (2015). ATM dependent silencing links nucleolar chromatin reorganization to DNA damage recognition. Cell Rep. 13, 251–259. doi: 10.1016/j.celrep.2015.08.085, PMID: 26440899 PMC4607662

[ref37] Harper L. Golubovskaya I. Cande W. Z. (2004). A bouquet of chromosomes. J. Cell Sci. 117, 4025–4032. doi: 10.1242/jcs.01363, PMID: 15316078

[ref38] Havlová K. Dvořáčková M. Peiro R. Abia D. Mozgová I. Vansáčová L.. (2016). Variation of 45S rDNA intergenic spacers in *Arabidopsis thaliana*. Plant Mol. Biol. 92, 457–471. doi: 10.1007/s11103-016-0524-1, PMID: 27531496

[ref39] Hays J. B. (2002). *Arabidopsis thaliana*, a versatile model system for study of eukaryotic genome-maintenance functions. DNA Repair 1, 579–600. doi: 10.1016/S1568-7864(02)00093-9, PMID: 12509283

[ref40] Horigome C. Unozawa E. Ooki T. Kobayashi T. (2019). Ribosomal RNA gene repeats associate with the nuclear pore complex for maintenance after DNA damage. PLoS Genet. 15:e1008103. doi: 10.1371/journal.pgen.1008103, PMID: 30998688 PMC6490929

[ref41] Hunter N. (2015). Meiotic recombination: the essence of heredity. Cold Spring Harb. Perspect. Biol. 7:a016618. doi: 10.1101/cshperspect.a016618, PMID: 26511629 PMC4665078

[ref42] Keller I. Chintauan-Marquier I. C. Veltsos P. Nichols R. A. (2006). Ribosomal DNA in the grasshopper *Podisma pedestris*: escape from concerted evolution. Genetics 174, 863–874. doi: 10.1534/genetics.106.061341, PMID: 16951064 PMC1602095

[ref43] Knoll A. Fauser F. Puchta H. (2014). DNA recombination in somatic plant cells: mechanisms and evolutionary consequences. Chromosom. Res. 22, 191–201. doi: 10.1007/s10577-014-9415-y, PMID: 24788060

[ref44] Krogh B. O. Symington L. S. (2004). Recombination proteins in yeast. Annu. Rev. Genet. 38, 233–271. doi: 10.1146/annurev.genet.38.072902.091500, PMID: 15568977

[ref45] Kuo B. A. Gonzalez I. L. Gillespie D. A. Sylvester J. E. (1996). Human ribosomal RNA variants from a single individual and their expression in different tissues. Nucleic Acids Res. 24, 4817–4824. doi: 10.1093/nar/24.23.4817, PMID: 8972871 PMC146304

[ref46] Kurylo C. M. Parks M. M. Juette M. F. Thibado J. K. Vincent C. T. Blanchard S. C.. (2018). Endogenous rRNA sequence variation can regulate stress response gene expression and phenotype article endogenous rRNA sequence variation can regulate stress response gene expression and phenotype. Cell Rep. 25, 236.e6–248.e6. doi: 10.1016/j.celrep.2018.08.093, PMID: 30282032 PMC6312700

[ref47] Layat E. Sáez-Vásquez J. Tourmente S. (2012). Regulation of pol I-transcribed 45S rDNA and pol III-transcribed 5S rDNA in arabidopsis. Plant Cell Physiol. 53, 267–276. doi: 10.1093/pcp/pcr177, PMID: 22173098

[ref48] Lees-Miller S. P. Meek K. (2003). Repair of DNA double strand breaks by non-homologous end joining. Biochimie 85, 1161–1173. doi: 10.1016/j.biochi.2003.10.011, PMID: 14726021

[ref49] Leigh J. W. Bryant D. (2015). POPART: full-feature software for haplotype network construction. Methods Ecol. Evol. 6, 1110–1116. doi: 10.1111/2041-210X.12410

[ref50] Leppek K. Fujii K. Quade N. Susanto T. T. Boehringer D. Lenarčič T.. (2020). Gene- and species-specific hox mRNA translation by ribosome expansion segments. Mol. Cell 80, 980.e13–995.e13. doi: 10.1016/j.molcel.2020.10.023, PMID: 33202249 PMC7769145

[ref51] Liu T. Huang J. (2014). Quality control of homologous recombination. Cell. Mol. Life Sci. 71, 3779–3797. doi: 10.1007/s00018-014-1649-5, PMID: 24858417 PMC11114062

[ref52] Lobrich M. Jeggo P. (2017). A process of resection-dependent nonhomologous end joining involving the goddess Artemis. Trends Biochem. Sci. 42, 690–701. doi: 10.1016/j.tibs.2017.06.011, PMID: 28739276 PMC5604544

[ref53] Long Q. Rabanal F. A. Meng D. Huber C. D. Farlow A. Platzer A.. (2013). Massive genomic variation and strong selection in *Arabidopsis thaliana* lines from Sweden. Nat. Genet. 45, 884–890. doi: 10.1038/ng.2678, PMID: 23793030 PMC3755268

[ref54] Lopez F. B. Fort A. Tadini L. Probst A. V. McHale M. Friel J.. (2021). Gene dosage compensation of rRNA transcript levels in *Arabidopsis thaliana* lines with reduced ribosomal gene copy number. Plant Cell 33, 1135–1150. doi: 10.1093/plcell/koab020, PMID: 33793816 PMC8225240

[ref55] Manova V. Gruszka D. (2015). DNA damage and repair in plants – from models to crops. Front. Plant Sci. 6:885. doi: 10.3389/fpls.2015.00885, PMID: 26557130 PMC4617055

[ref56] McVey M. Lee S. E. (2008). MMEJ repair of double-strand breaks (director’s cut): deleted sequences and alternative endings. Trends Genet. 24, 529–538. doi: 10.1016/j.tig.2008.08.007, PMID: 18809224 PMC5303623

[ref57] Meek K. Gupta S. Ramsden D. A. Lees-Miller S. P. (2004). The DNA-dependent protein kinase: the director at the end. Immunol. Rev. 200, 132–141. doi: 10.1111/j.0105-2896.2004.00162.x, PMID: 15242401

[ref58] Mentewab A. B. Jacobsen M. J. Flowers R. A. (2011). Incomplete homogenization of 18 S ribosomal DNA coding regions in *Arabidopsis thaliana*. BMC. Res. Notes 4:93. doi: 10.1186/1756-0500-4-93, PMID: 21453453 PMC3079661

[ref59] Mercier R. Mézard C. Jenczewski E. Macaisne N. Grelon M. Mezard C.. (2015). The molecular biology of meiosis in plants. Annu. Rev. Plant Biol. 66, 297–327. doi: 10.1146/annurev-arplant-050213-035923, PMID: 25494464

[ref60] Montacié C. Durut N. Opsomer A. Palm D. Comella P. Picart C.. (2017). Nucleolar proteome analysis and proteasomal activity assays reveal a link between nucleolus and 26S proteasome in *A. thaliana*. Front. Plant Sci. 8:1815. doi: 10.3389/fpls.2017.01815, PMID: 29104584 PMC5655116

[ref61] Moss T. Langlois F. Gagnon-Kugler T. Stefanovsky V. (2007). A housekeeper with power of attorney: the rRNA genes in ribosome biogenesis. Cell. Mol. Life Sci. 64, 29–49. doi: 10.1007/s00018-006-6278-1, PMID: 17171232 PMC11136161

[ref62] Mozgová I. Mokroš P. Fajkus J. (2010). Dysfunction of chromatin assembly factor 1 induces shortening of telomeres and loss of 45s rDNA in *Arabidopsis thaliana*. Plant Cell 22, 2768–2780. doi: 10.1105/tpc.110.076182, PMID: 20699390 PMC2947181

[ref63] Muchova V. Amiard S. Mozgova I. Dvorackova M. Gallego M. E. White C.. (2015). Homology-dependent repair is involved in 45S rDNA loss in plant CAF-1 mutants. Plant J. 81, 198–209. doi: 10.1111/tpj.12718, PMID: 25359579 PMC4309414

[ref64] Murata M. Heslop-Harrison J. S. Motoyoshi F. (1997). Physical mapping of the 5S ribosomal RNA genes in *Arabidopsis thaliana* by multi-color fluorescence in situ hybridization with cosmid clones. Plant J. 12, 31–37. doi: 10.1046/j.1365-313X.1997.12010031.x, PMID: 9263450

[ref65] Ohkura H. (2015). Meiosis: an overview of key differences from mitosis. Cold Spring Harb. Perspect. Biol. 7:a015859. doi: 10.1101/cshperspect.a015859, PMID: 25605710 PMC4448623

[ref66] Parks M. M. Kurylo C. M. Dass R. A. Bojmar L. Lyden D. Vincent C. T.. (2018). Variant ribosomal RNA alleles are conserved and exhibit tissue-specific expression. Sci. Adv. 4:eaao0665. doi: 10.1126/sciadv.aao0665, PMID: 29503865 PMC5829973

[ref67] Pavlistova V. Dvorackova M. Jez M. Mozgova I. Mokros P. Fajkus J. (2016). Phenotypic reversion in fas mutants of *Arabidopsis thaliana* by reintroduction of FAS genes: variable recovery of telomeres with major spatial rearrangements and transcriptional reprogramming of 45S rDNA genes. Plant J. 88, 411–424. doi: 10.1111/tpj.13257, PMID: 27377564

[ref68] Picart-Picolo A. Grob S. Picault N. Franek M. Llauro C. Halter T.. (2020). Large tandem duplications affect gene expression, 3D organization, and plant-pathogen response. Genome Res. 30, 1583–1592. doi: 10.1101/gr.261586.120, PMID: 33033057 PMC7605254

[ref69] Pillet L. Fontaine D. Pawlowski J. (2012). Intra-genomic ribosomal RNA polymorphism and morphological variation in elphidium macellum suggests inter-specific hybridization in Foraminifera. PLoS One 7:e32373. doi: 10.1371/journal.pone.0032373, PMID: 22393402 PMC3290570

[ref70] Polo S. E. Jackson S. P. (2011). Dynamics of DNA damage response proteins at DNA breaks: a focus on protein modifications. Genes Dev. 25, 409–433. doi: 10.1101/gad.2021311, PMID: 21363960 PMC3049283

[ref71] Pontvianne F. Abou-Ellail M. Douet J. Comella P. Matia I. Chandrasekhara C.. (2010). Nucleolin is required for DNA methylation state and the expression of rRNA gene variants in *Arabidopsis thaliana*. PLoS Genet. 6:e1001225. doi: 10.1371/journal.pgen.1001225, PMID: 21124873 PMC2991258

[ref72] Pontvianne F. Blevins T. Chandrasekhara C. Feng W. Stroud H. Jacobsen S. E.. (2012). Histone methyltransferases regulating rRNA gene dose and dosage control in Arabidopsis. Genes Dev. 26, 945–957. doi: 10.1101/gad.182865.111, PMID: 22549957 PMC3347792

[ref73] Pontvianne F. Blevins T. Chandrasekhara C. Mozgova I. Hassel C. Pontes O. M. F.. (2013). Subnuclear partitioning of rRNA genes between the nucleolus and nucleoplasm reflects alternative epiallelic states. Genes Dev. 27, 1545–1550. doi: 10.1101/gad.221648.113, PMID: 23873938 PMC3731543

[ref74] Rabanal F. A. Mandáková T. Soto-Jiménez L. M. Greenhalgh R. Parrott D. L. Lutzmayer S.. (2017a). Epistatic and allelic interactions control expression of ribosomal RNA gene clusters in *Arabidopsis thaliana*. Genome Biol. 18:75. doi: 10.1186/s13059-017-1209-z, PMID: 28464948 PMC5414317

[ref75] Rabanal F. A. Nizhynska V. Mandáková T. Novikova P. Y. Martin A. Lysak M. A.. (2017b). Unstable inheritance of 45S rRNA genes in *Arabidopsis thaliana*. G3 7, 1201–1209. doi: 10.1534/g3.117.040204, PMID: 28188182 PMC5386868

[ref76] Reynolds R. C. Montgomery P. O. B. Hughes B. (1964). Nucleolar “caps” produced by actinomycin D. Cancer Res. 24, 1269–1277. PMID: 14216161

[ref77] Ritossa F. M. Spiegelman S. (1965). Localization of DNA complementary to ribosomal RNA in the nucleolus organizer region of *Drosophila melanogaster*. Proc. Natl. Acad. Sci. U. S. A. 53, 737–745. doi: 10.1073/pnas.53.4.737, PMID: 14324529 PMC221060

[ref78] Robert T. Vrielynck N. Mézard C. de Massy B. Grelon M. Mezard C.. (2016). A new light on the meiotic DSB catalytic complex. Semin. Cell Dev. Biol. 54, 165–176. doi: 10.1016/j.semcdb.2016.02.025, PMID: 26995551

[ref79] Rohrig S. Schropfer S. Knoll A. Puchta H. Röhrig S. Schröpfer S.. (2016). The RTR complex partner RMI2 and the DNA helicase RTEL1 are both independently involved in preserving the stability of 45S rDNA repeats in *Arabidopsis thaliana*. PLoS Genet. 12:e1006394. doi: 10.1371/journal.pgen.1006394, PMID: 27760121 PMC5070779

[ref80] Roitinger E. Hofer M. Kocher T. Pichler P. Novatchkova M. Yang J.. (2015). Quantitative phosphoproteomics of the ATM and ATR dependent DNA damage response in *Arabidopsis thaliana*. Mol. Cell. Proteomics 14, 556–571. doi: 10.1074/mcp.M114.040352, PMID: 25561503 PMC4349977

[ref81] San-Segundo P. A. Clemente-Blanco A. (2020). Resolvases, dissolvases, and helicases in homologous recombination: clearing the road for chromosome segregation. Genes 11:71. doi: 10.3390/genes11010071, PMID: 31936378 PMC7017083

[ref82] Sasaki M. Lange J. Keeney S. (2010). Genome destabilization by homologous recombination in the germ line. Nat. Rev. Mol. Cell Biol. 11, 182–195. doi: 10.1038/nrm2849, PMID: 20164840 PMC3073813

[ref83] Seol J. H. Shim E. Y. Lee S. E. (2018). Microhomology-mediated end joining: good, bad and ugly. Mutat. Res. 809, 81–87. doi: 10.1016/j.mrfmmm.2017.07.002, PMID: 28754468 PMC6477918

[ref84] Simon L. Rabanal F. A. Dubos T. Oliver C. Lauber D. Poulet A.. (2018). Genetic and epigenetic variation in 5S ribosomal RNA genes reveals genome dynamics in *Arabidopsis thaliana*. Nucleic Acids Res. 46, 3019–3033. doi: 10.1093/nar/gky163, PMID: 29518237 PMC5887818

[ref85] Sims J. Copenhaver G. P. Schlögelhofer P. (2019). Meiotic DNA repair in the nucleolus employs a nonhomologous end-joining mechanism. Plant Cell 31, 2259–2275. doi: 10.1105/tpc.19.00367, PMID: 31266898 PMC6751124

[ref86] Sims J. Sestini G. Elgert C. von Haeseler A. Schlögelhofer P. (2021). Sequencing of the Arabidopsis NOR2 reveals its distinct organization and tissue-specific rRNA ribosomal variants. Nat. Commun. 12:387. doi: 10.1038/s41467-020-20728-6, PMID: 33452254 PMC7810690

[ref87] Sluis M. V. McStay B. van Sluis M. McStay B. (2015). A localized nucleolar DNA damage response facilitates recruitment of the homology-directed repair machinery independent of cell cycle stage. Genes Dev. 29, 1151–1163. doi: 10.1101/gad.260703.115, PMID: 26019174 PMC4470283

[ref88] Song W. Joo M. Yeom J. H. Shin E. Lee M. Choi H. K.. (2019). Divergent rRNAs as regulators of gene expression at the ribosome level. Nat. Microbiol. 4, 515–526. doi: 10.1038/s41564-018-0341-1, PMID: 30718849

[ref89] Stage D. E. Eickbush T. H. (2007). Sequence variation within the rRNA gene loci of 12 Drosophila species. Genome Res. 17, 1888–1897. doi: 10.1101/gr.6376807, PMID: 17989256 PMC2099596

[ref90] Sung P. Krejci L. Van Komen S. Sehorn M. G. (2003). Rad51 recombinase and recombination mediators. J. Biol. Chem. 278, 42729–42732. doi: 10.1074/jbc.R300027200, PMID: 12912992

[ref91] Takeuchi Y. Horiuchi T. Kobayashi T. (2003). Transcription-dependent recombination and the role of fork collision in yeast rDNA. Genes Dev. 17, 1497–1506. doi: 10.1101/gad.1085403, PMID: 12783853 PMC196080

[ref92] Templeton A. R. Crandall K. A. Sing C. F. (1992). A cladistic analysis of phenotypic associations with haplotypes inferred from restriction endonuclease mapping. III. Cladogram estimation. Genetics 132, 619–633. doi: 10.1093/genetics/132.2.619, PMID: 1385266 PMC1205162

[ref93] Templeton G. W. Moorhead G. B. (2005). The phosphoinositide-3-OH-kinase-related kinases of *Arabidopsis thaliana*. EMBO Rep. 6, 723–728. doi: 10.1038/sj.embor.7400479, PMID: 16065066 PMC1369146

[ref94] Tseng H. Chou W. Wang J. Zhang X. Zhang S. Schultz R. M. (2008). Mouse ribosomal RNA genes contain multiple differentially regulated variants. PLoS One 3:e1843. doi: 10.1371/journal.pone.0001843, PMID: 18365001 PMC2266999

[ref95] van Sluis M. McStay B. (2017). Nucleolar reorganization in response to rDNA damage. Curr. Opin. Cell Biol. 46, 81–86. doi: 10.1016/j.ceb.2017.03.004, PMID: 28431265

[ref96] Vesper O. Amitai S. Belitsky M. Byrgazov K. Kaberdina A. C. Engelberg-Kulka H.. (2011). Selective translation of leaderless mRNAs by specialized ribosomes generated by MazF in *Escherichia coli*. Cell 147, 147–157. doi: 10.1016/j.cell.2011.07.047, PMID: 21944167 PMC4894548

[ref97] Wallace H. Birnstiel M. L. (1966). Ribosomal cistrons and the nucleolar organizer. Biochim. Biophys. Acta 114, 296–310. doi: 10.1016/0005-2787(66)90311-X, PMID: 5943882

[ref98] Wang M. Wu W. Wu W. Rosidi B. Zhang L. Wang H.. (2006). PARP-1 and Ku compete for repair of DNA double strand breaks by distinct NHEJ pathways. Nucleic Acids Res. 34, 6170–6182. doi: 10.1093/nar/gkl840, PMID: 17088286 PMC1693894

[ref99] Warmerdam D. O. O. van den Berg J. Medema R. H. H. van den Berg J. Medema R. H. H. van den Berg J.. (2016). Breaks in the 45S rDNA lead to recombination-mediated loss of repeats. Cell Rep. 14, 2519–2527. doi: 10.1016/j.celrep.2016.02.048, PMID: 26972008

[ref100] Warner J. R. (1999). The economics of ribosome biosynthesis in yeast. Trends Biochem. Sci. 24, 437–440. doi: 10.1016/S0968-0004(99)01460-7, PMID: 10542411

[ref101] Waters A. P. Syin C. McCutchan T. F. (1989). Developmental regulation of stage-specific ribosome populations in Plasmodium. Nature 342, 438–440. doi: 10.1038/342438a0, PMID: 2586613

[ref102] Wright W. D. Shah S. S. Heyer W. D. (2018). Homologous recombination and the repair of DNA double-strand breaks. J. Biol. Chem. 293, 10524–10535. doi: 10.1074/jbc.TM118.000372, PMID: 29599286 PMC6036207

[ref103] Wu Y. Gao T. Wang X. Hu Y. Hu X. Hu Z.. (2014). TALE nickase mediates high efficient targeted transgene integration at the human multi-copy ribosomal DNA locus. Biochem. Biophys. Res. Commun. 446, 261–266. doi: 10.1016/j.bbrc.2014.02.099, PMID: 24589733

[ref104] Xu Y. Xu D. (2020). Repair pathway choice for double-strand breaks. Essays Biochem. 64, 765–777. doi: 10.1042/EBC20200007, PMID: 32648897

[ref105] Yoshiyama K. O. (2016). Recent progress in research on DNA damage responsesin animals and plants. Genes Genet. Syst. 90, 185–186. doi: 10.1266/ggs.15-10001, PMID: 26616757

[ref106] Yoshiyama K. O. Sakaguchi K. Kimura S. (2013). DNA damage response in plants: conserved and variable response compared to animals. Biology 2, 1338–1356. doi: 10.3390/biology2041338, PMID: 24833228 PMC4009792

