# Supplementary figures and images for: It Is Just a Matter of Time: Balancing Homologous Recombination and Non-homologous End Joining at the rDNA Locus During Meiosis
Source: Front Plant Sci. 2021 Oct 28;12:773052. doi: 10.3389/fpls.2021.773052 (PMC8580885; doi:10.3389/fpls.2021.773052)

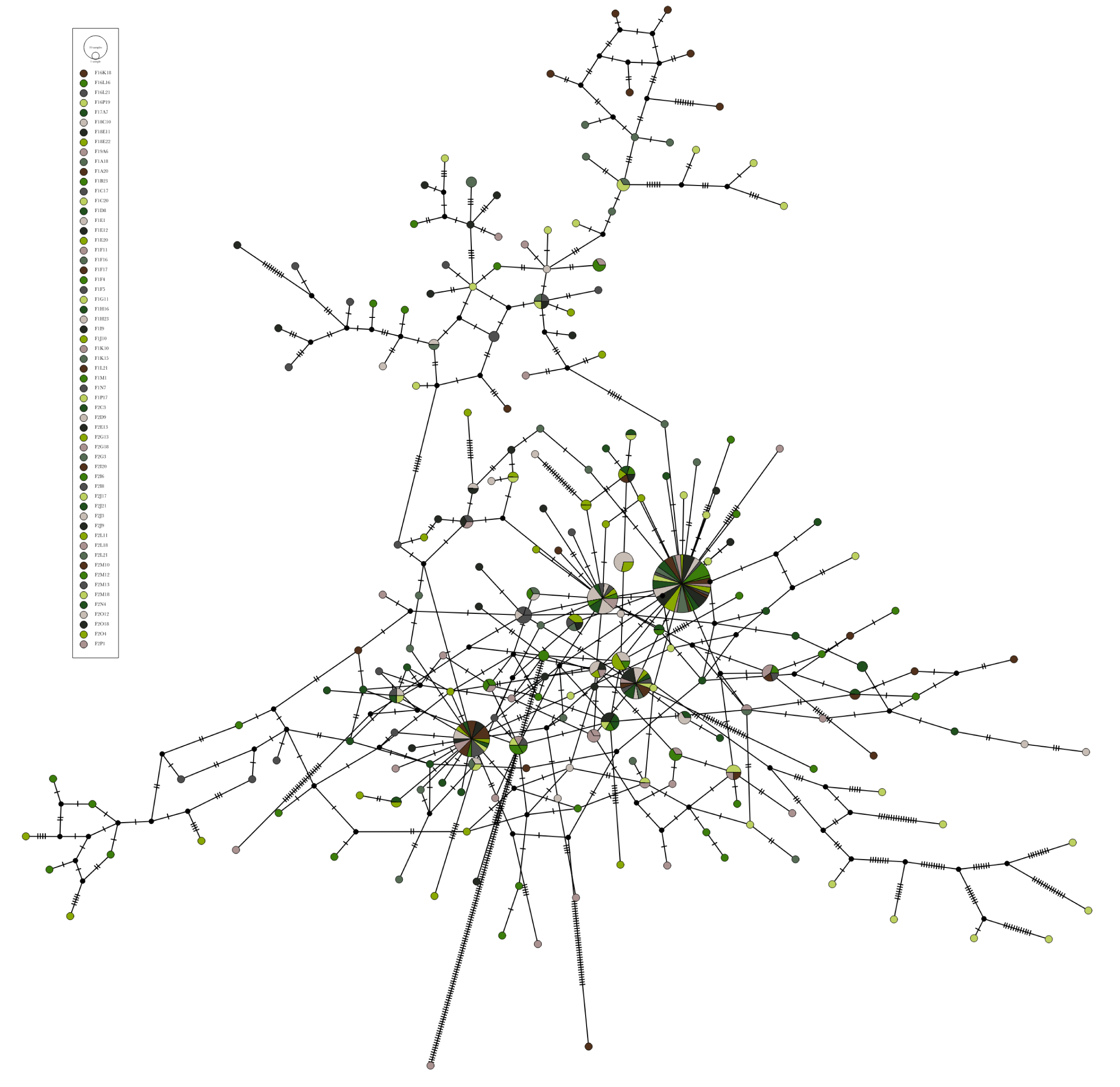

Supplement: Supplementary file 1 [file Image_1.TIF]
